# Impact of SARS-CoV-2 Spike Mutations on Its Activation by TMPRSS2 and the Alternative TMPRSS13 Protease

**DOI:** 10.1128/mbio.01376-22

**Published:** 2022-08-01

**Authors:** Annelies Stevaert, Ria Van Berwaer, Cato Mestdagh, Julie Vandeput, Els Vanstreels, Valerie Raeymaekers, Manon Laporte, Lieve Naesens

**Affiliations:** a KU Leuven, Department of Microbiology, Immunology and Transplantation, Rega Institute, Leuven, Belgium; b Department of Microbiology, Icahn School of Medicine at Mount Sinai, New York, New York, USA; c Global Health and Emerging Pathogens Institute, Icahn School of Medicine at Mount Sinai, New York, New York, USA; University of Hong Kong

**Keywords:** SARS-CoV-2, human coronavirus 229E, spike protein, mutation, protease, cleavage, TMPRSS2, TMPRSS13

## Abstract

The continuous emergence of new variants of severe acute respiratory syndrome coronavirus 2 (SARS-CoV-2) urges better understanding of the functional motifs in the spike (S) protein and their tolerance to mutations. Here, we focused on the S2′ motif, which, during virus entry, requires cleavage by a host cell protease to release the fusion peptide. Though belonging to an immunogenic region, the SARS-CoV-2 S2′ motif (811-KPSKR-815) has shown hardly any variation, with its three basic (K/R) residues being >99.99% conserved thus far. By creating a series of mutant pseudoviruses bearing the spikes of Wuhan-Hu-1, its G614 mutant or the Delta and Omicron variants, we show that residue K_814_ (preceding the scissile R_815_) is dispensable for TMPRSS2 yet favored by the alternative TMPRSS13 protease. Activation by TMPRSS13 was drastically reduced when the SARS-CoV-2 S2′ motif was swapped with that of the low pathogenic 229E coronavirus (685-RVAGR-689), and also, the reverse effect was seen. This swap had no impact on recognition by TMPRSS2. In the Middle East respiratory syndrome coronavirus (MERS-CoV) spike, introducing a dibasic scissile motif was easily accepted by TMPRSS13 but less so by TMPRSS2, confirming that TMPRSS13 favors a sequence rich in K/R residues. Pseudovirus entry experiments in Calu-3 cells confirmed that the S2′ mutations have minor impact on TMPRSS2. Our findings are the first to demonstrate which S2′ residues are important for SARS-CoV-2 spike activation by these two airway proteases, with TMPRSS2 being more tolerant to variation than TMPRSS13. This preemptive insight will help to estimate the impact of S2′ motif changes as they appear in new SARS-CoV-2 variants.

## OBSERVATION

**FIG 1:**
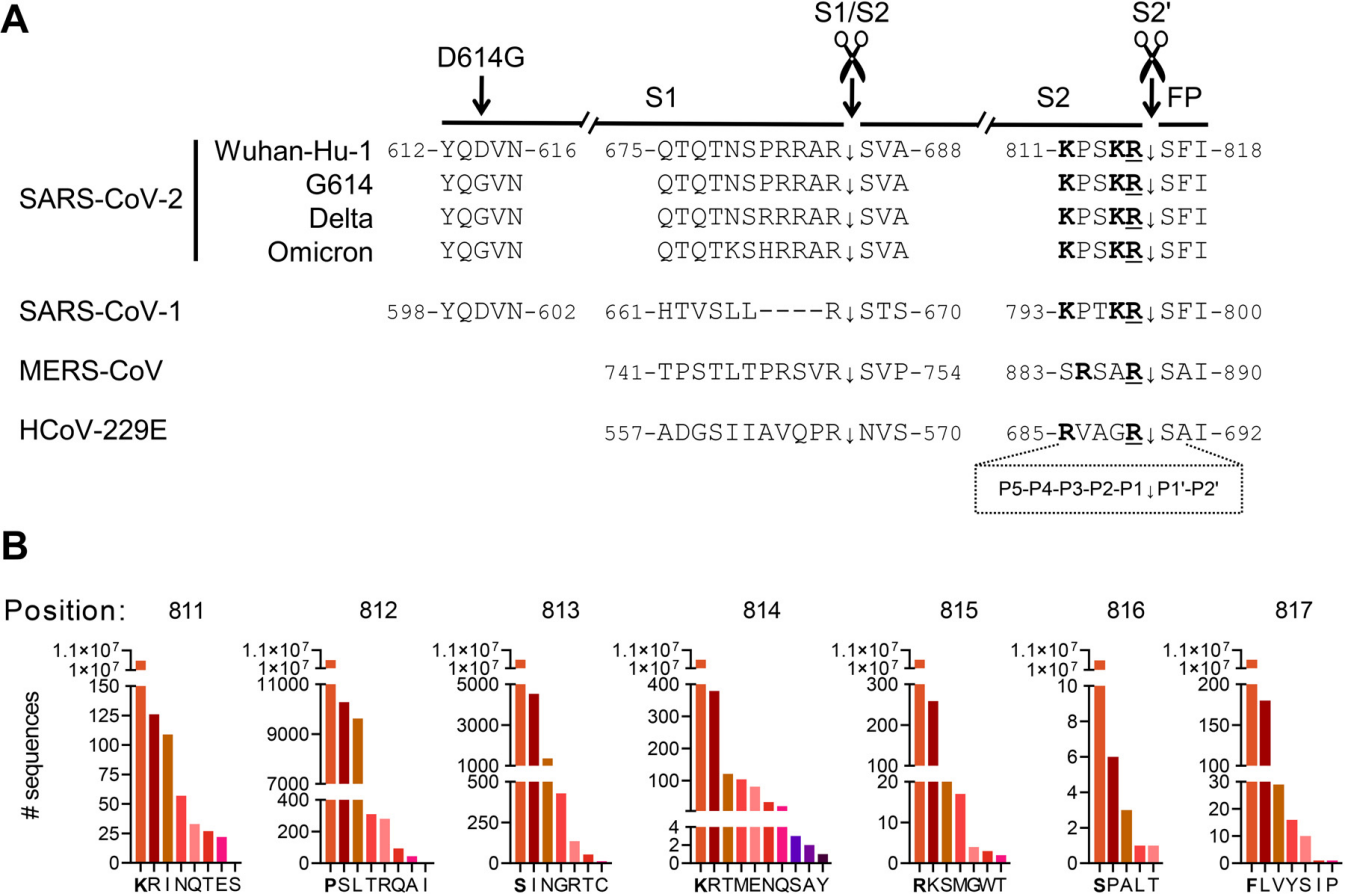
The S2′ motif of SARS-CoV-2 is rich in basic residues and, so far, highly conserved. (A) The S2′ motif is identical in the Wuhan-Hu-1 strain and G614, Delta, and Omicron variants of SARS-CoV-2 and conserved in SARS-CoV-1. Its KR motif is not present in MERS-S and 229E-S, while the scissile R (underlined) is shared by all spikes. Boldface indicates basic (R or K) residues. FP, fusion peptide. The alignment further shows the sequence differences in the S1/S2 and D614 regions (note that amino acid differences outside these regions are not covered). The frame at the bottom shows the P5-P1 and P1′-P2′ designation of the S2′ cleavage motif. (B) To determine the conservation rate of the P5 to P2′ residues, a variation analysis was performed on 10,480,461 SARS-2-S sequences submitted to the GISAID database between 10 January 2020 and 29 April 2022. The *y* axis (different scales in the various panels) shows the number of sequences carrying the specified amino acid, with the consensus residue marked in bold on the *x* axis.

Since its emergence in December 2019, SARS-CoV-2 is undergoing worldwide selection, with frequent appearance of new variants. The variability in the viral spike (S) antigen is linked to immune evasion but also affects the functioning of S in virus entry, replication, and transmission. Substitution D614G, which arose in March 2020 and soon became dominant, increases S protein stability (1, 2) and adoption of the open spike conformation, promoting virus transmission (3, 4). Mutation P681R is present in the Delta and Kappa variants and located adjacent to the S1/S2 furin recognition motif (RRAR) (Fig. 1A). This mutation enhances cleavage of full-length S to the S1/S2 subunit form (5), the primed state that provokes cell-cell fusion (6). Accordingly, P681R-mutant viruses like the Delta variant exhibit higher fusogenicity, more efficient replication in cell culture (7), and higher pathogenicity in hamsters (5). Conversely, the Omicron variant has a less fusogenic S protein and markedly lower virulence (8). Despite three mutations near the S1/S2 site (Fig. 1A), the spikes of Omicron virions are mostly unprimed (9). Hence, the virulence and transmissibility of SARS-CoV-2 are linked to delicately tuned cleavage characteristics of its spike protein.

Whereas S1/S2 priming is not required to allow virus entry, cleavage of the S2′ site (Fig. 1A) is essential, since it releases the fusion peptide (FP). In case of SARS-CoV-2 and MERS-CoV, S2′ cleavage occurs at or near the cell surface (10) when the virion is abundant in S1/S2 primed spikes. S2′ cleavage of the SARS-CoV-2 spike is also promoted by engagement of the ACE2 receptor (11). When priming is inefficient (e.g., in Omicron), the virus favors the alternative endosomal route in which the spike is activated by cathepsin B/L (9, 12). Regarding the cell surface route, the key role of the TMPRSS2 protease in SARS-CoV-2 infection was established *in vitro* (13) and *in vivo*. In a SARS-CoV-2 mouse model, TMPRSS2 knockout led to a significant reduction in lung pathology and virus titers (14). Still, the observation that replication was not abrogated implies that S can be activated by alternative airway proteases. One candidate, TMPRSS13, activates SARS-CoV-2 S quite effectively (2, 15, 16). In human airway-derived Calu-3 cells, TMPRSS2 knockdown reduced virus replication dramatically (2, 17), yet TMPRSS13 knockdown also had a significant effect (2). For TMPRSS2, but not TMPRSS13, the importance was confirmed in human airway organoids (18). Hence, the role of TMPRSS13 in SARS-CoV-2 infection remains unclear.

Knowing which residues in the S1/S2 and S2′ cleavage motifs are essential can help to assess the impact of new variations as they emerge. Whereas the above-cited studies looked at S1/S2 site variations, we focused here on the S2′ motif. Intrigued by the observation that a dibasic scissile motif is present in SARS-CoV-2 but lacking in all four low-pathogenicity human coronaviruses, we created a series of S-pseudotyped viruses bearing mutations at P2-P5 (i.e., residues preceding the scissile R) (Fig. 1A). The efficiency of TMPRSS2 and TMPRSS13 to activate these S2′ mutants was assessed in transfected HEK293T cells and Calu-3 cells, a cell line with endogenous expression of TMPRSS2 and TMPRSS13. We verified that the S2′ mutations had no impact on expression and priming of the spike in pseudoparticles. Based on our findings, we conclude that TMPRSS2 readily accepts variations at the S2′ motif, whereas TMPRSS13 is more fastidious, with a preference for K/R-rich motifs such as those present in SARS-CoV-2 but missing in less pathogenic coronaviruses.

### The S2′ site of SARS-CoV-2 shows low variability and K/R abundance.

Within the SARS-CoV-2 S2′ motif (811-KPSKR-815), the scissile R_815_ residue is flanked by a second basic (K_814_) residue (Fig. 1A). The motif lies in a strong B-cell epitope that elicits antibodies with various virus-neutralizing capacity (19, 20), and for which the titers seem correlated with COVID-19 disease severity (21). Longitudinal analysis of sera from vaccinated and infected individuals identified several potential sites for antibody escape mutations in the FP epitope region (22), including residues K_811_, K_814_, and R_815_ in the S2′ motif. Still, in terms of virus evolution, the S2′ motif has so far shown strikingly low diversity (23). When we analyzed the ~10.5 million spike sequences in the GISAID database (Fig. 1B), all three basic residues in the S2′ motif (K_811_, K_814_, and R_815_) proved highly dominant, being present in >99.99% of the sequences. This is less surprising for R_815_ (in only a few cases substituted by K), since it is the probable scissile residue (11, 24). P_812_ and S_813_ are somewhat more tolerant to variation, consistent with the presence of T_813_ in the SARS-CoV-1 spike (Fig. 1A) (25).

### Mutations at the S2′ motif do not affect spike expression or S1/S2 cleavage.

The high conservation of the SARS-CoV-2 KxxKR motif suggests that its sequence is essential for spike activation. Strikingly, a dibasic scissile motif is missing in all four endemic, less pathogenic human coronaviruses (HCoVs), including HCoV-229E (25), while MERS-CoV bears an xRxxR motif that can be processed by furin (26) (Fig. 1A). Hence, we designed a series of S2′-mutated S proteins (abbreviated SARS-2-S, MERS-S, and 229E-S) in which we swapped the motifs from SARS-2-S and 229E-S or introduced or removed a Lys (K) at P2 or an Arg (R) at P4 (since MERS-S contains a basic residue at P4 but not P5). For SARS-2-S, the mutations were introduced into the spikes of Wuhan-Hu-1, its G614 mutant, the Delta variant, and the Omicron BA.1 variant.

**FIG 2:**
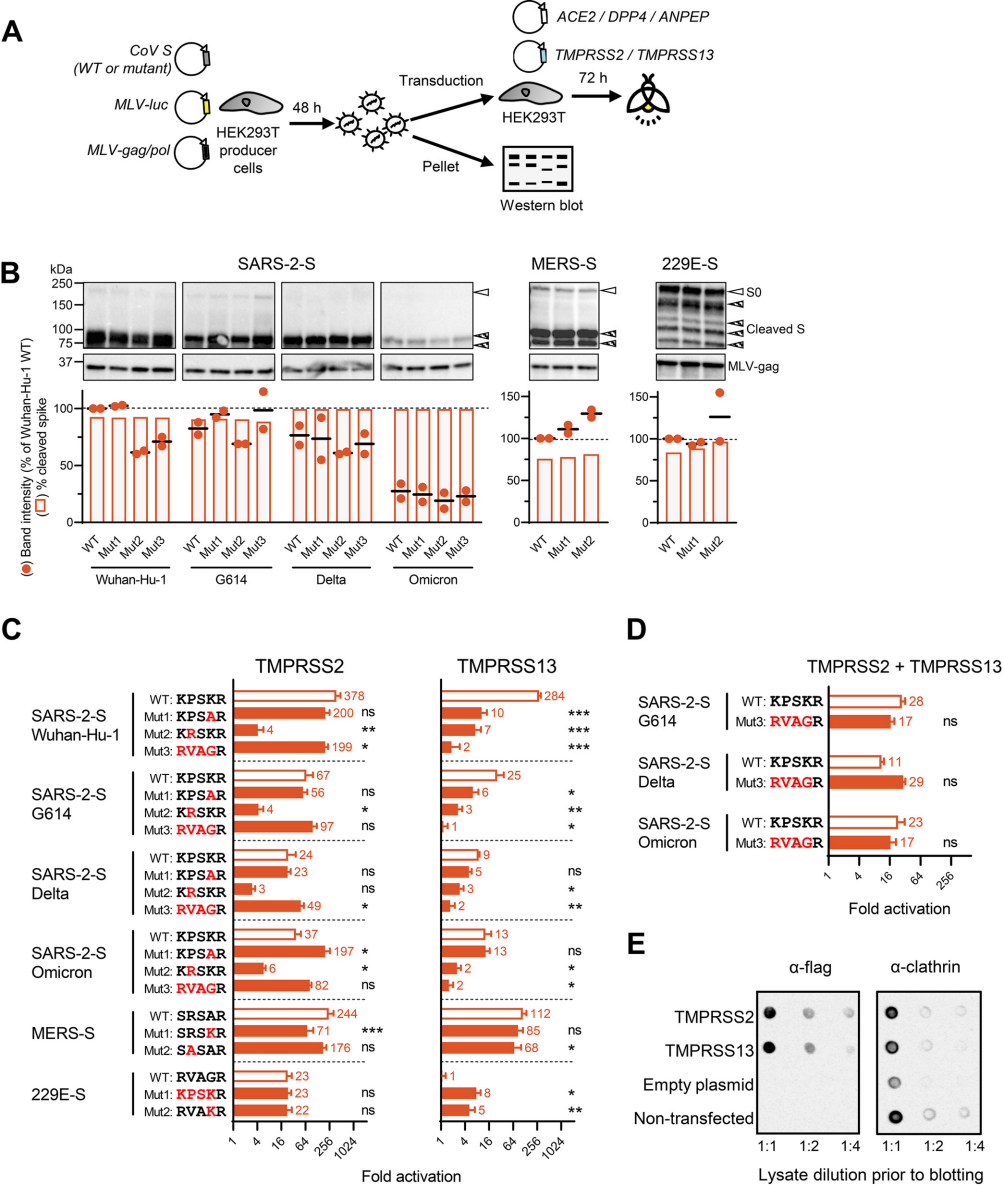
Impact of S2′ site changes on TMPRSS2- or TMPRSS13-mediated activation of pseudovirus entry. (A) Experimental setup. (B) Spike protein levels and cleavage state in S-pseudotyped virions. The particles were produced in HEK293T cells, pelleted, and subjected to western blot analysis with anti-V5 antibody recognizing the S0 and S2 forms (top) and anti-MLV gag antibody (bottom). Open arrowheads, full-length (S0) spike; hatched arrowheads, cleaved forms resulting from S1/S2 processing (SARS-2-S and MERS-S) or less specific cleavage (229E-S). The graphs below the images show the quantitative data based on band intensities, obtained for two batches of pseudoparticles. Circles, sum of all S bands (normalized to the band of MLV-gag), expressed relative to Wuhan-Hu-1 WT for SARS-2-S and the respective WT for MERS-S and 229E-S; bars, percent cleaved spike, calculated as 100 − [S0]/[sum of all S bands]. (C) Entry of WT and mutant S2′ S-pseudotyped viruses into HEK293T cells transfected with TMPRSS2 or TMPRSS13. The bars show the factor activation, i.e., luminescence signal relative to the condition receiving empty instead of protease plasmid. Pooled data from four experiments, each performed in five or six replicates, are shown. *, *P* ≤ 0.05; **, *P* ≤ 0.01; ***, *P* ≤ 0.001 (nested *t* test, two tailed). ns, not significant. (D) Entry activation in TMPRSS2/TMPRSS13-cotransfected HEK293T cells, for the S2′-WT and -Mut3 forms of G614, Delta, and Omicron SARS-2-S pseudoviruses. ns, not significant (*P* > 0.05) (E). Expression of TMPRSS2 and TMPRSS13 following transfection in HEK293T cells. The two Flag-tagged proteases were produced at similar protein levels, as assessed by dot blot assay with anti-Flag antibody.

Two separate batches of S-bearing MLV-pseudoparticles were produced in HEK293T cells, pelleted, and analyzed for spike levels and cleavage by western blotting (Fig. 2A). Overall, the S2′-mutant pseudovirions were found to carry levels of total S protein (Fig. 2B, circles) and cleaved S (Fig. 2B, bars) comparable to those of their respective wild-type (WT) viruses. Even Mut3, which carries a change of 4 of 5 residues (RVAGR), showed spike expression and S1/S2 cleavage similar to those of the WT. For the four variants of SARS-2-S, band intensity tended to be slightly lower for Mut2 (KRSKR). The total S levels were comparable for the S2′-WT pseudovirions of Wuhan-Hu-1, G614, and Delta but 4-fold lower in the Omicron variant, which equals the reduction seen in another study (9). Uncleaved S0 accounted for ~8% of the total S protein level in Wuhan-Hu-1 and G614 and was not detected in Delta and Omicron particles (Fig. 2B, bars). The superior S1/S2 priming of the Delta spike is well documented (5, 7). Also, the S2′ mutants of MERS-S showed S1/S2 priming equally efficient (~80%) to that of the WT. For 229E-S, the WT and mutants contained similar levels of several cleavage products. In short, the level and priming state of pseudovirion spikes proved unaffected by the S2′ site changes that we studied.

### Introducing the 229E-S2′ motif in SARS-2-S causes severe impairment for TMPRSS13 but not for TMPRSS2.

We next conducted a virus entry assay to monitor S2′ activation by TMPRSS2 and TMPRSS13. Pseudoviruses carrying WT or mutant S2′ spikes were transduced into HEK293T cells which had been transfected 1 day earlier with TMPRSS2, TMPRSS13, or empty (control) plasmid, plus the cognate virus receptor (Fig. 2A). Transduction was performed in the presence of E64d to shut off the endosomal route in which S is activated by cathepsin B/L instead of cell surface proteases (27).

TMPRSS2 and TMPRSS13 proved efficient activators of the four SARS-2-S variants, giving an increase in S-driven pseudovirus entry (compared to cells transfected with empty plasmid) between 9-fold (TMPRSS13 for Delta-WT) and 378-fold (TMPRSS2 for Wuhan-Hu-1-WT) (Fig. 2C). As expected (2, 13, 28), TMPRSS2 effectively activated the three CoV species, the fold increase being 378, 244, and 23 for the WT forms of SARS-2-S Wuhan-Hu-1, MERS-S, and 229E-S, respectively. The lower value for 229E-S is explained by lower levels of functional S in these pseudovirions, due to unspecific cleavage (see above). Importantly, 229E-S was not activated by TMPRSS13, in sharp contrast with efficient usage of this protease by SARS-2-S and MERS-S.

For SARS-2-S, TMPRSS2 activation was not or only slightly affected when the basic charge at P2 (K to A; Mut1) was removed or the S2′ motif was swapped with that of 229E-S (Mut3). The effect of these mutations was also variable: compared to the respective WT, Mut3 of Wuhan-Hu-1 showed a minor reduction (2-fold; *P* = 0.023), while the Delta-Mut3 and Omicron-Mut1 pseudoviruses showed a slight increase in TMPRSS2 activation (2-fold [*P* = 0.022] and 5-fold [*P* = 0.037], respectively). In stark contrast, Mut1 and particularly Mut3 had consistently negative impact on TMPRSS13-driven entry. For the Wuhan-Hu-1 spike, the reduction versus WT was 27-fold (Mut1) and 155-fold (Mut3) (*P* < 0.001 in both cases). The impairment by Mut3 was less dramatic but still significant for the G614, Delta, and Omicron variants (fold reduction versus their WT: 22 [*P* = 0.011]; 5 [*P* = 0.0080] and 8 [*P* = 0.014], respectively). Hence, introducing the 229E-S2′ motif into SARS-2-S impaired the capacity of TMPRSS13 to activate this spike. Inversely, 229E-S gained the ability to use TMPRSS13 for entry activation when bearing the SARS-2-S2′ motif (Mut1; 7-fold; *P* = 0.044) or its K at P2 (Mut2; 5-fold; *P* = 0.0076).

All four SARS-2-S Mut2 (KRSKR) spikes showed significantly lower activation by TMPRSS2 and TMPRSS13. Since this mutated S2′ motif contains a polybasic furin recognition motif (KRxKR), Mut2 may possibly be more subject to extracellular furin (2), making it less dependent on TMPRSS2 or TMPRSS13. This agrees with a report showing enhanced furin cleavage for KRRKR-mutant SARS-2-S protein (29). Our assumption is supported by the observation that MERS-S became 3-fold less TMPRSS2 dependent when its minimal furin recognition motif (RxxR in WT) (26) was rendered more basic (RxKR in Mut1). Still, the picture may be quite complex, since the inverse change, i.e., removing the P4-Arg residue that is required for furin recognition (SASAR; Mut2), did not lead to higher activation of MERS-S by TMPRSS2 or TMPRSS13.

Finally, we assessed the entry of SARS-2-S Mut3 pseudovirions in cells transfected with a combination of the TMPRSS2 and TMPRSS13 plasmids (Fig. 2D). Since the Mut3 viruses now showed entry comparable to that of their WT (*P* > 0.2 for the three variant pairs tested), TMPRSS2 proved able to successfully compensate for their severe impairment for TMPRSS13.

In short, substituting the P2-basic residue or the P2-P5 motif of SARS-2-S had a minor influence on pseudovirus activation by TMPRSS2 but a significant impact on that by TMPRSS13, a protease that disfavors the 229E-S2′ motif. The impairment of these S2′ mutants for TMPRSS13 is not visible in cells that coexpress the more efficient and less discriminating protease TMPRSS2.

### The expressed TMPRSS2 and TMPRSS13 proteases show a similar pattern of autocleavage and subcellular localization.

Considering that low expression of TMPRSS13 might be the reason for poor activation of some pseudoviruses, we verified the protease expression level under our assay conditions. When transfected into HEK293T cells in equal DNA amounts, the TMPRSS2 and TMPRSS13 plasmids generated comparable levels of Flag-tagged protein, as evident from dot blot analysis with anti-Flag antibody (Fig. 2E). In addition, we did western blotting with antibodies specific for the extracellular serine protease region of TMPRSS2 and intracellular region of TMPRSS13 (Fig. S1A). In the case of TMPRSS2, the polyclonal antibody detected two specific bands besides a few aspecific products. The band of ~55 kDa represents full-length TMPRSS2 (30), while that of ~32 kDa represents the serine protease domain resulting from autocleavage. For TMPRSS13, we observed two dominant bands: full-length TMPRSS13 having, in glycosylated form, a molecular weight (MW) of ~70 kDa and the presumed intracellular fragment of ~26 kDa that results from autocleavage (31).

10.1128/mbio.01376-22.1 FIG S1 Expression, cleavage and localization of TMPRSS2 and TMPRSS13 following transfection in HEK293T cells. (A) Western blot analysis with antibodies directed to the serine protease domain of TMPRSS2 and intracellular region of TMPRSS13. Besides full-length TMPRSS2 and TMPRSS13 (open arrows), autocleavage products (dashed arrows) were prominently present, indicating efficient protease activity. (B) Confocal microscopy on transfected 293AD cells, stained with anti-Flag tag antibody under cell-permeabilized conditions. Bar, 10 μm. Download FIG S1, PDF file, 0.9 MB. Copyright © 2022 Stevaert et al. 2022 Stevaert et al. https://creativecommons.org/licenses/by/4.0/ This content is distributed under the terms of the Creative Commons Attribution 4.0 International license.

Next, to examine the subcellular localization of the expressed proteases, we performed immunostaining of the transfected cells followed by confocal microscopy. While the TMPRSS2- and TMPRSS13-specific antibodies yielded a negative result, anti-Flag detection under cell-permeabilized conditions resulted in clear and specific staining (Fig. S1B). TMPRSS2 and TMPRSS13 showed a strikingly similar staining pattern, with weak staining of the plasma membrane and apparently high abundance in the endoplasmic reticulum (ER) and secretory pathway. The same picture was seen by another group, who confirmed the ER localization of expressed TMPRSS13 by costaining with an anti-ER antibody (31). We observed no plasma membrane staining under nonpermeabilized conditions, in full accordance with another study using a C-terminus (in this case V5 tag)-directed antibody (32). The authors observed that the cell surface localization was promoted by cotransfecting an inhibitory protein that suppresses autocleavage of TMPRSS13.

To conclude, our pseudovirus entry experiments were conducted under conditions where both proteases were efficiently produced in their active form. The expressed TMPRSS2 and TMPRSS13 proteins showed intriguing resemblance in terms of subcellular localization.

### The S2′ mutations only slightly affect virus entry in Calu-3 cells, in which SARS-2-S is activated by TMPRSS2.

Since the above results were obtained under conditions of protease overexpression, we also determined the entry efficiency of the S2′-mutant SARS-2-S pseudoviruses in Calu-3 cells, which endogenously express TMPRSS2 and TMPRSS13 as well as the ACE2 receptor for virus attachment (2). Also, these experiments were conducted in the presence of the cathepsin inhibitor E64d.

**FIG 3:**
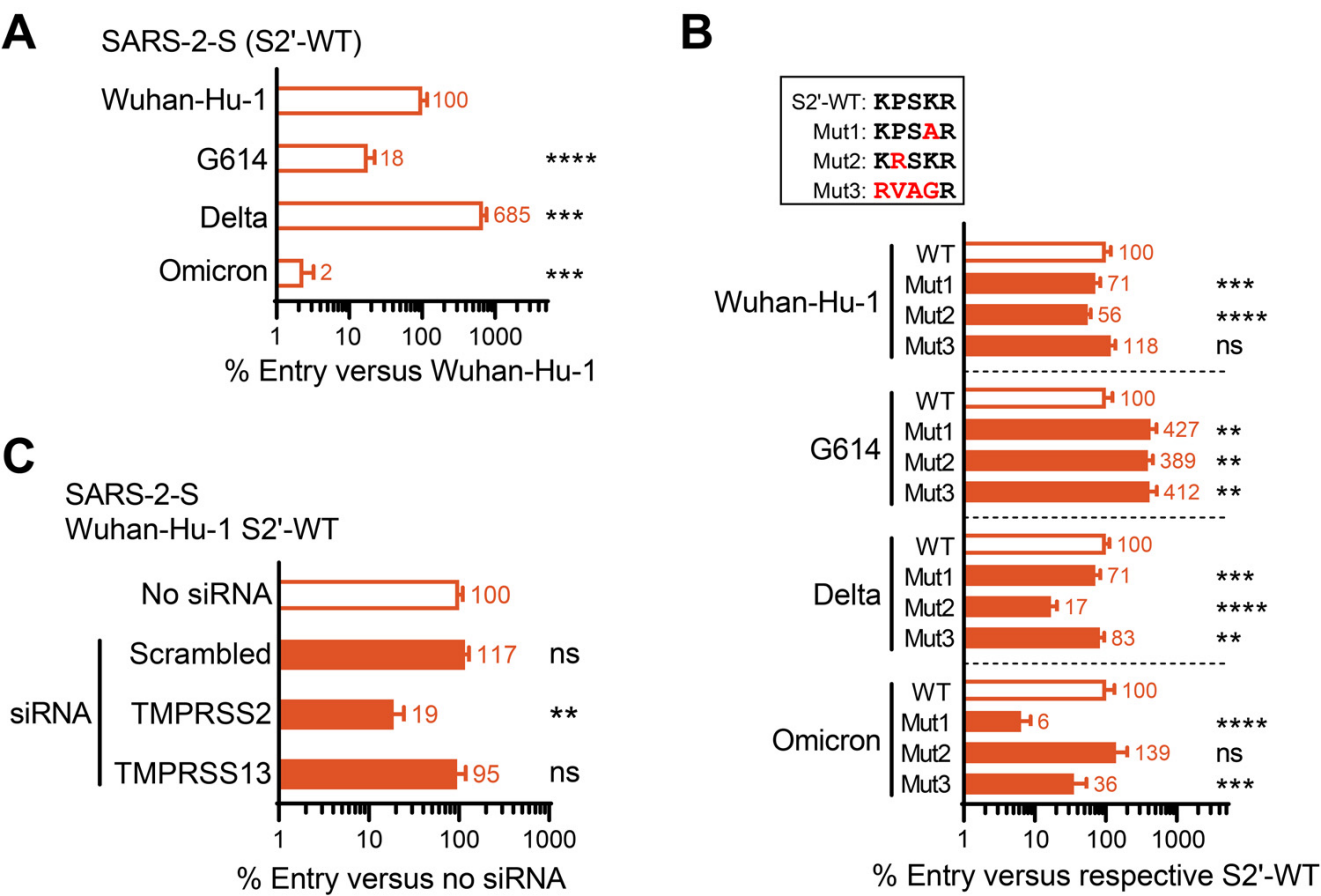
Impact of the S2′ motif changes on SARS-2-S pseudovirus entry in Calu-3 cells, in which TMPRSS2 is the activating protease. (A) Entry efficiency of the four S2′-WT viruses, expressed relative to Wuhan-Hu-1 pseudovirus. (B) Entry of the S2′-mutants, expressed relative to the corresponding WT. (A and B) Pooled data from three experiments, each performed in 6 replicates. (C) Knocking down TMPRSS2 in Calu-3 cells caused a significant reduction in the entry efficiency of Wuhan-Hu-1 pseudovirus (S2′-WT), while knockdown of TMPRSS13 had no effect. Mean results of two experiments, each performed in three to four replicates, are shown. **, *P* ≤ 0.01; ***, *P* ≤ 0.001; ****, *P* ≤ 0.0001 (nested *t* test, two-tailed). ns, not significant.

Regarding the S2′-WT forms, Delta pseudovirus entered Calu-3 cells 7-fold more efficiently than its Wuhan-Hu-1 counterpart (Fig. 3A), while Omicron showed a dramatic reduction of 43-fold. These findings concur with other reports (9, 33). The G614 variant exhibited 5-fold-lower Calu-3 cell entry than its D614 (i.e., Wuhan-Hu-1) parent. This can be explained by the S-protein-stabilizing effect of mutation D614G, leading to higher reliance on the cathepsin route (2), which we inhibited by conducting the assay in the presence of E64d.

For most S2′ mutants (Fig. 3B), the differences with their WT were small, even though they were statistically significant due to very low variability in the Calu-3 entry assay. The profile was similar for the Wuhan-Hu-1 and Delta pseudovirions, with Mut2 giving the strongest reduction, Mut1 being slightly reduced and Mut3 being barely affected. The mutants’ profile was different for the two variants exhibiting reduced Calu-3 cell entry and higher use of the cathepsin route. Consequently, the correlation between the Calu-3 cell results and those obtained in transfected HEK293T cells was clearest for Wuhan-Hu-1 and Delta, the two SARS-2-S variants that are very effective at using the TMPRSS2 route. The furin-promoting S2′ motif in Mut2 reduced the use of TMPRSS2 in both cell lines, but the effect was less pronounced in Calu-3 cells, which express rather low levels of furin (M. Laporte, unpublished data). Entry of the Mut3 forms of Wuhan-Hu-1 and Delta was only slightly affected in Calu-3 and TMPRSS2-transfected HEK293T cells, in stark contrast to the severe impairment of Mut3 in TMPRSS13-transfected HEK293T cells. This validates that TMPRSS2 is the main activator of SARS-2-S in Calu-3 cells.

Along the same line, siRNA-mediated knockdown of TMPRSS2 in Calu-3 cells caused a significant reduction of 81%, in the entry efficiency of SARS-2-S pseudovirus (Wuhan-Hu-1 S2′-WT) (Fig. 3C). Knocking down TMPRSS13 had no effect. Since the knockdown conditions were optimized for Calu-3 cells (34) and validated for these two proteases (2), we conclude that TMPRSS2 overrules TMPRSS13 in activating the spike in these cells.

### Broader relevance of our findings.

This study anticipates the potential impact of S2′ site changes as they may appear in new SARS-CoV-2 variants. Since the S2′ motif lies in a strong B-cell epitope (19–21), the virus might acquire mutations in this region to escape the antibody response (22, 23). The fact that such variations are, thus far, rarely detected suggests that the sequence of this K/R-rich motif is crucial for SARS-2-S activation by cell surface proteases. Our study is the first to address this topic. First of all, we show that TMPRSS2 readily accepts changes in the sequence preceding the scissile Arg (R_815_), in line with its ability to activate different coronavirus spikes but also the fusion proteins of influenza A and B virus, parainfluenza virus, and metapneumovirus (35), which all have different P2-P5 sequences. The diversity of TMPRSS2 in terms of P2-P4 recognition was also visible in a broad analysis of its substrate specificity (36). Reciprocally, we found that TMPRSS13 is more restricted, showing preference for a dibasic scissile motif and aversion of the HCoV-229E P2-P5 sequence. The basic P2 residue (K_814_) differentiates SARS-2-S from 229E-S and the spikes of all other low pathogenic HCoVs, which otherwise share a basic P5 residue. For each of the four SARS-2-S variants that we studied (including the omicron variant, which is peculiar in strongly relying on cathepsin-mediated endosomal entry [9, 12]), introducing the 229E-S2′ motif caused severe impairment of TMPRSS13 cleavability. Since the reverse effect was also seen, we propose that recognition by TMPRSS13 might be one of the factors regulating coronavirus virulence. In fact, the preference of TMPRSS13 for K/R-rich cleavage motifs concurs with its activity on multibasic hemagglutinins of highly pathogenic avian influenza viruses (37). Combined with the presence of TMPRSS13 in human lung tissue (38), this raises the hypothesis that loss of TMPRSS13 cleavability, by mutation of K_814_ or other unfavorable changes at P2-P5, might reduce the capacity of SARS-CoV-2 to replicate in the lungs.

The key role of TMPRSS2 in SARS-CoV-2 infection has been established by knockdown studies in a mouse model (14), human airway organoids (18), and the Calu-3 cell model (2, 17). Multicycle replication of SARS-CoV-2 in Calu-3 cells was reduced by about 90% and 40% after knockdown of TMPRSS2 and TMPRSS13, respectively (2). The pseudovirus entry data in this report confirm that TMPRSS2 surpasses TMPRSS13 as an activator of SARS-2-S. This was evident in Calu-3 cells but even more so in HEK293T cells that we cotransfected for both proteases. To fully assess the relevance of TMPRSS13, a cell type would be needed that expresses TMPRSS13 but not TMPRSS2, besides the ACE2 receptor. To address this, we performed an analysis of open access single-cell RNA sequencing data (Fig. S2 to S4) (39–44). *TMPRSS13* transcripts were found to be prominent in club cells and secretory cells of human bronchus tissue and in alveolar type 1 and type 2 cells of human lung tissue (Fig. S2). Coexpression of *TMPRSS13* and *ACE2* was visible in nasal goblet and ciliated cells, bronchial club cells, and ileum enterocytes from healthy donors (Fig. S3), as well as in goblet, club and secretory cells from airway samples of COVID-19 patients (Fig. S4). In other tissue samples (from healthy donors), coexpression was apparent only for *TMPRSS2* and *ACE2*, i.e., in lung alveolar cells, in enterocytes and progenitor cells from colon and rectum, and in kidney proximal tubule cells. In pancreas and liver cells, neither TMPRSS13 nor TMPRSS2 was coexpressed with ACE2. Although this analysis may contradict an important role for TMPRSS13 in SARS-CoV-2 infection, a subtle effect in specific cell types (potentially bearing a virus receptor other than ACE2) remains possible and is worth further investigation. For instance, to complement our pseudovirus experiments, a cell-cell fusion assay could help to unravel the interplay between the spike (in the S1/S2-primed or unprimed state), its receptor, and S2′-activating protease and assess whether TMPRSS2 and TMPRSS13 may differ in this regard. In this context, we refer to the link between spike fusogenicity and SARS-CoV-2 virulence, observed in animal studies with the Delta and Omicron variants (5, 7, 8). Second, we are puzzled by the observation that the TMPRSS2 and TMPRSS13 proteins expressed in HEK293T cells gave relatively weak staining of the plasma membrane but appeared to localize mainly in the ER and secretory pathway (as already seen by others [31, 45]). Since both undergo (auto)cleavage and shedding of the extracellular protease domain, it remains to be determined in which form these proteases conduct S2′ cleavage during virus entry.

10.1128/mbio.01376-22.2 FIG S2 Expression of TMPRSS13 in different cell types of human bronchus (left) and lung (right) tissue. The images were made by the open access tools available via (A) Human Protein Atlas (proteinatlas.org) and (B) https://digital.bihealth.org. Panel B shows single-cell RNA sequencing analyses from the work of Lukassen et al. (39) for cells derived from subsegmental bronchial branches (HBEC, human bronchial epithelial cells) (left) and lung tissue samples from nonsmoking and smoking donors combined (right). Download FIG S2, PDF file, 2.1 MB. Copyright © 2022 Stevaert et al. 2022 Stevaert et al. https://creativecommons.org/licenses/by/4.0/ This content is distributed under the terms of the Creative Commons Attribution 4.0 International license.

10.1128/mbio.01376-22.3 FIG S3 Correlation graphs for TMPRSS13, TMPRSS2, and ACE2 expression in tissue samples from healthy donors. The dots are colored according to the cell type, specified left of the graphs. These single-cell RNA sequencing analyses are described in the following references: Vieira Braga et al. (40), Wang et al. (41), Baron et al. (42), MacParland et al. (43) and Stewart et al. (44). Graphs were created with https://www.covid19cellatlas.org from Cellxgene Data Portal (Chanzuckerberg Initiative; https://cellxgene.cziscience.com/). Download FIG S3, PDF file, 1.8 MB. Copyright © 2022 Stevaert et al. 2022 Stevaert et al. https://creativecommons.org/licenses/by/4.0/ This content is distributed under the terms of the Creative Commons Attribution 4.0 International license.

10.1128/mbio.01376-22.4 FIG S4 Correlation graphs for TMPRSS13, TMPRSS2, and ACE2 expression in airway tissue samples from COVID-19 patient donors. Graphs were created with https://www.covid19cellatlas.org from Cellxgene Data Portal (Chanzuckerberg Initiative; https://cellxgene.cziscience.com/); the dots are colored according to the cell type, specified left of the graphs. Download FIG S4, PDF file, 1.0 MB. Copyright © 2022 Stevaert et al. 2022 Stevaert et al. https://creativecommons.org/licenses/by/4.0/ This content is distributed under the terms of the Creative Commons Attribution 4.0 International license.

To conclude, though limited to a few mutations, our study shows that TMPRSS2 easily accepts variations in the SARS-2-S2′ motif, whereas the alternative TMPRSS13 protease strongly favors a K/R-rich motif. This knowledge will help to estimate the impact of S2′ site changes as soon as they are observed during variant surveillance.

### Experimental procedures.

**(i) Plasmid material.** The SARS-2-S sequences were cloned in the pCAGGS vector, starting from codon-optimized cDNAs for the spike of Wuhan-Hu-1 (reference sequence YP_009724390.1), an Omicron BA.1 strain (GenBank no. UFO69279.1; B.1.1.529) (both donated by K. Dallmeier, Leuven, Belgium), or a Delta strain (GISAID number: EPI_ISL_12180364) (pLV-SpikeV8 from InvivoGen). The plasmids encoding MERS-S (reference sequence YP_009047204.1) and 229E-S (reference sequence NP_073551.1) were a kind gift from S. Pöhlmann (Göttingen, Germany). All spike sequences contained the authentic ER retention signal (which was introduced if missing in the original cDNA) and a V5 tag added at the C terminus. Cloning and introduction of mutations (i.e., D614G and S2′ site changes) were performed with overlapping primers and NEBuilder HiFi DNA Assembly.

The expression plasmids for human angiotensin-converting enzyme 2 (ACE2; receptor for SARS-2-S); dipeptidyl peptidase-4 (DPP4; for MERS-S); and aminopeptidase N (APN; for 229E-S), were donated by S. Pöhlmann. The pcDNA3.1+/C-(K)DYK-based plasmids encoding human TMPRSS2 and TMPRSS13 with a C-terminal Flag tag were purchased from GenScript (34).

**(ii) Pseudovirus entry assay in HEK293T and Calu-3 cells.** Detailed methods can be found elsewhere (2). Briefly, to produce S-pseudotyped murine leukemia virus (MLV) particles, the S-plasmids were combined with MLV gag-pol and firefly luciferase reporter plasmids (a gift from S. Pöhlmann) and forward transfected into HEK293T cells (Thermo Scientific no. HCL4517), using Lipofectamine 2000 reagent (Invitrogen) and a 6-well format. After 3 days incubation at 33°C (SARS-2-S and 229E-S) or 37°C (MERS-S), the pseudovirus-containing supernatants were clarified by centrifugation and stored in aliquots at −80°C.

Activation of pseudovirus entry by TMPRSS2 and TMPRSS13 was evaluated in white collagen-coated half-area 96-well plates, using medium with 10% fetal calf serum (FCS) during all cell incubations. On day −1, Fugene 6 reagent (Promega; 0.1 μL per well) was used for reverse transfection of HEK293T cells (15,000 cells per well) with TMPRSS2, TMPRSS13, or empty plasmid combined with ACE2, DPP4, or APN plasmid (5 ng of each DNA per well; reduced to 2.5 ng of TMPRSS2 plasmid and TMPRSS13 plasmid in the cotransfection experiment). After overnight incubation at 37°C, the cells were first preincubated for 2 h with 50 μM E64d. Next, the WT and mutant S2′ pseudoviruses were added and allowed to enter for 2 h. After replacement of the medium, the plates were incubated for 3 days at 33°C. Finally, luciferase signal was measured using a luciferase assay system kit and a GloMax Navigator luminometer (both from Promega). The assays were conducted four times with each condition tested in five or six replicate wells.

To assess pseudovirus entry into Calu-3 cells (ATCC HTB-55), the cells were seeded in half-area 96-well plates at 30,000 cells per well. One day later, they were exposed to 40 μM E64d for 20 min at 37°C. After pseudovirus addition, entry was promoted by 45 min spinoculation at 453 × *g* and 37°C, after which the plates were incubated for 2 h at 37°C. Throughout virus entry, the medium contained low protein content (0.2% FCS and 0.03% bovine serum albumin [BSA]) to keep the cell surface proteases fully active. Next, the inoculum was replaced by medium with 10% FCS and the plates were incubated for 3 days at 33°C. Luminescence was recorded as above. The Calu-3 assays were conducted three times with each condition tested in six replicate wells.

For the knockdown study in Calu-3 cells (see reference 34 for all details), cell suspensions were placed in white 96-well plates at 35,000 cells per well and then reverse transfected with 10 nM small interfering RNA (siRNA) for TMPRSS2 or TMPRSS13 or a scrambled control (On-TARGETplus siRNA SMARTpools from Dharmacon), using 0.5 μL per well of Lipofectamine RNAiMAX (Thermo Fisher Scientific). One day later, the transfection medium containing 10% FCS was replaced by medium with 0.2% FCS. At day 2 posttransfection, the above-described procedure was used for pseudovirus transduction, followed by luminescence recording after 3 days incubation. The knockdown experiment was conducted twice in three or four replicate wells.

**(iii) Western blot and dot blot analyses.** To collect the pseudovirions, the supernatant stocks were added on top of a 20% sucrose cushion and centrifuged (2 h at 21,000 × *g* and 4°C). After the pellet was lysed in radioimmunoprecipitation assay (RIPA) buffer, the lysates were mixed with reducing loading dye, boiled, and then resolved on Bio-Rad XT precast 4 to 12% bis-Tris gels using XT-MOPS (morpholinepropanesulfonic acid) running buffer. After blotting, the membranes were stained with anti-V5 (Invitrogen R960-25; 1:1,000) and anti-MLV gag p30 (Abcam ab130757; 1:1,000) antibodies combined with horseradish peroxidase (HRP)-linked secondary antibody (Dako P0447; 1:4,000). The bands were detected with SuperSignal West Femto substrate (Thermo Scientific), imaged on a Bio-Rad ChemiDoc MP imaging apparatus, and quantified with Image Lab software. Spike band intensities were expressed relative to the band of MLV gag.

To analyze protease expression, HEK293T cells were transfected for TMPRSS2 and TMPRSS13 (using the same conditions as in the pseudovirus assay) and, 48 h later, subjected to RIPA extraction. After denaturing PAGE as described above, the blots were stained with antibodies against TMPRSS2 (Life Technologies PA5-14265; 1:500), TMPRSS13 (Abcam Ab59862; 1:1,000), and clathrin (BD Biosciences 610499; 1:1,000), combined with HRP-linked secondary antibodies (Dako P0399 [1:4,000] and P0447 [1:8,000]). The bands were detected and imaged as above. For the dot blot analysis, 2 μL of each HEK293T extract was spotted on nitrocellulose membranes in undiluted or ½- and 1/4-diluted forms. Detection with anti-Flag tag (Abcam Ab205606; 1:500), anticlathrin, and secondary antibodies was carried out as described above.

**(iv) Confocal microscopy.** To circumvent cell loss during multiple wash steps, these experiments were done with 293AD cells (Cell Biolabs AD-100), a HEK293-derived cell line selected for superior plate attachment. The cells were transfected with TMPRSS2 or TMPRSS13 as described above and seeded at 45,000 cells per well in 8-well plastic chamber slides (Ibidi). Two days later, the cells were fixed with 4% formaldehyde and permeabilized with 0.1% Triton X-100, with phosphate-buffered saline (PBS) washes between. Next, the cells were stained with anti-Flag tag antibody (Abcam Ab205606; 1:280) and Alexa Fluor 488-labeled goat anti-rabbit antibody (Thermo Fisher Scientific A-11008; 1:500). The antibody diluent, 3% BSA in PBS, was also used in the wash steps. Last, the cell nuclei were stained with Hoechst-33342 (Invitrogen). Microscopy was performed on a TCS SP5 confocal microscope (Leica Microsystems). All images were captured with an HCX PL APO 63 (numerical aperture [NA], 1.2) water immersion objective.

**(v) Analysis of SARS-CoV-2 spike variance.** A total of 10,480,461 spike sequences were downloaded from the GISAID database (46) on 9 May 2022 and converted into a Basic Local Alignment Search Tool (BLAST) database with CLC Main Workbench (Qiagen). Each possible variant sequence carrying a single substitution at amino acids 811 to 817 was then used as a BLAST query.

**(vi) Statistical analysis.** The values for averages and standard deviations were calculated from pooled luminescence readouts from three or four experiments. The statistical significance of differences between two data sets was analyzed by two-tailed nested *t* test, using GraphPad Prism v.9.3.1 software.

## References

[1] Zhang L, Jackson CB, Mou H, Ojha A, Peng H, Quinlan BD, Rangarajan ES, Pan A, Vanderheiden A, Suthar MS, Li W, Izard T, Rader C, Farzan M, Choe H. 2020. SARS-CoV-2 spike-protein D614G mutation increases virion spike density and infectivity. Nat Commun 11:6013. doi:10.1038/s41467-020-19808-4. 33243994 PMC7693302

[2] Laporte M, Raeymaekers V, Van Berwaer R, Vandeput J, Marchand-Casas I, Thibaut HJ, Van Looveren D, Martens K, Hoffmann M, Maes P, Pöhlmann S, Naesens L, Stevaert A. 2021. The SARS-CoV-2 and other human coronavirus spike proteins are fine-tuned towards temperature and proteases of the human airways. PLoS Pathog 17:e1009500. doi:10.1371/journal.ppat.1009500. 33886690 PMC8061995

[3] Hou YJ, Chiba S, Halfmann P, Ehre C, Kuroda M, Dinnon KH, 3rd, Leist SR, Schafer A, Nakajima N, Takahashi K, Lee RE, Mascenik TM, Graham R, Edwards CE, Tse LV, Okuda K, Markmann AJ, Bartelt L, de Silva A, Margolis DM, Boucher RC, Randell SH, Suzuki T, Gralinski LE, Kawaoka Y, Baric RS. 2020. SARS-CoV-2 D614G variant exhibits efficient replication ex vivo and transmission in vivo. Science 370:1464–1468. doi:10.1126/science.abe8499. 33184236 PMC7775736

[4] Zhou B, Thao TTN, Hoffmann D, Taddeo A, Ebert N, Labroussaa F, Pohlmann A, King J, Steiner S, Kelly JN, Portmann J, Halwe NJ, Ulrich L, Trueb BS, Fan X, Hoffmann B, Wang L, Thomann L, Lin X, Stalder H, Pozzi B, de Brot S, Jiang N, Cui D, Hossain J, Wilson MM, Keller MW, Stark TJ, Barnes JR, Dijkman R, Jores J, Benarafa C, Wentworth DE, Thiel V, Beer M. 2021. SARS-CoV-2 spike D614G change enhances replication and transmission. Nature 592:122–127. doi:10.1038/s41586-021-03361-1. 33636719

[5] Saito A, Irie T, Suzuki R, Maemura T, Nasser H, Uriu K, Kosugi Y, Shirakawa K, Sadamasu K, Kimura I, Ito J, Wu J, Iwatsuki-Horimoto K, Ito M, Yamayoshi S, Loeber S, Tsuda M, Wang L, Ozono S, Butlertanaka EP, Tanaka YL, Shimizu R, Shimizu K, Yoshimatsu K, Kawabata R, Sakaguchi T, Tokunaga K, Yoshida I, Asakura H, Nagashima M, Kazuma Y, Nomura R, Horisawa Y, Yoshimura K, Takaori-Kondo A, Imai M, Tanaka S, Nakagawa S, Ikeda T, Fukuhara T, Kawaoka Y, Sato K, Genotype to Phenotype Japan Consortium. 2022. Enhanced fusogenicity and pathogenicity of SARS-CoV-2 Delta P681R mutation. Nature 602:300–306. doi:10.1038/s41586-021-04266-9. 34823256 PMC8828475

[6] Papa G, Mallery DL, Albecka A, Welch LG, Cattin-Ortolá J, Luptak J, Paul D, McMahon HT, Goodfellow IG, Carter A, Munro S, James LC. 2021. Furin cleavage of SARS-CoV-2 spike promotes but is not essential for infection and cell-cell fusion. PLoS Pathog 17:e1009246. doi:10.1371/journal.ppat.1009246. 33493182 PMC7861537

[7] Mlcochova P, Kemp SA, Dhar MS, Papa G, Meng B, Ferreira I, Datir R, Collier DA, Albecka A, Singh S, Pandey R, Brown J, Zhou J, Goonawardane N, Mishra S, Whittaker C, Mellan T, Marwal R, Datta M, Sengupta S, Ponnusamy K, Radhakrishnan VS, Abdullahi A, Charles O, Chattopadhyay P, Devi P, Caputo D, Peacock T, Wattal C, Goel N, Satwik A, Vaishya R, Agarwal M, Mavousian A, Lee JH, Bassi J, Silacci-Fegni C, Saliba C, Pinto D, Irie T, Yoshida I, Hamilton WL, Sato K, Bhatt S, Flaxman S, James LC, Corti D, Piccoli L, Barclay WS, Rakshit P, CITIID-NIHR BioResource COVID-19 Collaboration, et al. 2021. SARS-CoV-2 B.1.617.2 Delta variant replication and immune evasion. Nature 599:114–119. doi:10.1038/s41586-021-03944-y. 34488225 PMC8566220

[8] Halfmann PJ, Iida S, Iwatsuki-Horimoto K, Maemura T, Kiso M, Scheaffer SM, Darling TL, Joshi A, Loeber S, Singh G, Foster SL, Ying B, Case JB, Chong Z, Whitener B, Moliva J, Floyd K, Ujie M, Nakajima N, Ito M, Wright R, Uraki R, Warang P, Gagne M, Li R, Sakai-Tagawa Y, Liu Y, Larson D, Osorio JE, Hernandez-Ortiz JP, Henry AR, Ciuoderis K, Florek KR, Patel M, Odle A, Wong LR, Bateman AC, Wang Z, Edara VV, Chong Z, Franks J, Jeevan T, Fabrizio T, DeBeauchamp J, Kercher L, Seiler P, Gonzalez-Reiche AS, Sordillo EM, Chang LA, van Bakel H, Consortium Mount Sinai Pathogen Surveillance (PSP) study group, et al. 2022. SARS-CoV-2 Omicron virus causes attenuated disease in mice and hamsters. Nature 603:687–692. doi:10.1038/s41586-022-04441-6. 35062015 PMC8942849

[9] Meng B, Abdullahi A, Ferreira I, Goonawardane N, Saito A, Kimura I, Yamasoba D, Gerber PP, Fatihi S, Rathore S, Zepeda SK, Papa G, Kemp SA, Ikeda T, Toyoda M, Tan TS, Kuramochi J, Mitsunaga S, Ueno T, Shirakawa K, Takaori-Kondo A, Brevini T, Mallery DL, Charles OJ, Bowen JE, Joshi A, Walls AC, Jackson L, Martin D, Smith KGC, Bradley J, Briggs JAG, Choi J, Madissoon E, Meyer KB, Mlcochova P, Ceron-Gutierrez L, Doffinger R, Teichmann SA, Fisher AJ, Pizzuto MS, de Marco A, Corti D, Hosmillo M, Lee JH, James LC, Thukral L, Veesler D, Sigal A, Sampaziotis F, Ecuador-COVID19 Consortium, et al. 2022. Altered TMPRSS2 usage by SARS-CoV-2 Omicron impacts infectivity and fusogenicity. Nature 603:706–714. doi:10.1038/s41586-022-04474-x. 35104837 PMC8942856

[10] Koch J, Uckeley ZM, Doldan P, Stanifer M, Boulant S, Lozach PY. 2021. TMPRSS2 expression dictates the entry route used by SARS-CoV-2 to infect host cells. EMBO J 40:e107821. doi:10.15252/embj.2021107821. 34159616 PMC8365257

[11] Yu S, Zheng X, Zhou B, Li J, Chen M, Deng R, Wong G, Lavillette D, Meng G. 2022. SARS-CoV-2 spike engagement of ACE2 primes S2' site cleavage and fusion initiation. Proc Natl Acad Sci USA 119:e2111199119. doi:10.1073/pnas.2111199119. 34930824 PMC8740742

[12] Zhao H, Lu L, Peng Z, Chen LL, Meng X, Zhang C, Ip JD, Chan WM, Chu AW, Chan KH, Jin DY, Chen H, Yuen KY, To KK. 2022. SARS-CoV-2 Omicron variant shows less efficient replication and fusion activity when compared with Delta variant in TMPRSS2-expressed cells. Emerg Microbes Infect 11:277–283. doi:10.1080/22221751.2021.2023329. 34951565 PMC8774049

[13] Hoffmann M, Kleine-Weber H, Schroeder S, Krüger N, Herrler T, Erichsen S, Schiergens TS, Herrler G, Wu NH, Nitsche A, Muller MA, Drosten C, Pohlmann S. 2020. SARS-CoV-2 cell entry depends on ACE2 and TMPRSS2 and is blocked by a clinically proven protease inhibitor. Cell 181:271–280.E8. doi:10.1016/j.cell.2020.02.052. 32142651 PMC7102627

[14] Li F, Han M, Dai P, Xu W, He J, Tao X, Wu Y, Tong X, Xia X, Guo W, Zhou Y, Li Y, Zhu Y, Zhang X, Liu Z, Aji R, Cai X, Li Y, Qu D, Chen Y, Jiang S, Wang Q, Ji H, Xie Y, Sun Y, Lu L, Gao D. 2021. Distinct mechanisms for TMPRSS2 expression explain organ-specific inhibition of SARS-CoV-2 infection by enzalutamide. Nat Commun 12:866. doi:10.1038/s41467-021-21171-x. 33558541 PMC7870838

[15] Hoffmann M, Hofmann-Winkler H, Smith JC, Kruger N, Arora P, Sorensen LK, Sogaard OS, Hasselstrom JB, Winkler M, Hempel T, Raich L, Olsson S, Danov O, Jonigk D, Yamazoe T, Yamatsuta K, Mizuno H, Ludwig S, Noe F, Kjolby M, Braun A, Sheltzer JM, Pohlmann S. 2021. Camostat mesylate inhibits SARS-CoV-2 activation by TMPRSS2-related proteases and its metabolite GBPA exerts antiviral activity. EBioMedicine 65:103255. doi:10.1016/j.ebiom.2021.103255. 33676899 PMC7930809

[16] Kishimoto M, Uemura K, Sanaki T, Sato A, Hall WW, Kariwa H, Orba Y, Sawa H, Sasaki M. 2021. TMPRSS11D and TMPRSS13 activate the SARS-CoV-2 spike protein. Viruses 13:384. doi:10.3390/v13030384. 33671076 PMC8001073

[17] Bestle D, Heindl MR, Limburg H, Van Lam van T, Pilgram O, Moulton H, Stein DA, Hardes K, Eickmann M, Dolnik O, Rohde C, Klenk H-D, Garten W, Steinmetzer T, Böttcher-Friebertshäuser E. 2020. TMPRSS2 and furin are both essential for proteolytic activation of SARS-CoV-2 in human airway cells. Life Sci Alliance 3:e202000786. doi:10.26508/lsa.202000786. 32703818 PMC7383062

[18] Beumer J, Geurts MH, Lamers MM, Puschhof J, Zhang J, van der Vaart J, Mykytyn AZ, Breugem TI, Riesebosch S, Schipper D, van den Doel PB, de Lau W, Pleguezuelos-Manzano C, Busslinger G, Haagmans BL, Clevers H. 2021. A CRISPR/Cas9 genetically engineered organoid biobank reveals essential host factors for coronaviruses. Nat Commun 12:5498. doi:10.1038/s41467-021-25729-7. 34535662 PMC8448725

[19] Poh CM, Carissimo G, Wang B, Amrun SN, Lee CY, Chee RS, Fong SW, Yeo NK, Lee WH, Torres-Ruesta A, Leo YS, Chen MI, Tan SY, Chai LYA, Kalimuddin S, Kheng SSG, Thien SY, Young BE, Lye DC, Hanson BJ, Wang CI, Renia L, Ng LFP. 2020. Two linear epitopes on the SARS-CoV-2 spike protein that elicit neutralising antibodies in COVID-19 patients. Nat Commun 11:2806. doi:10.1038/s41467-020-16638-2. 32483236 PMC7264175

[20] Vanderheijden N, Stevaert A, Xie J, Ren X, Barbezange C, Noppen S, Desombere I, Verhasselt B, Geldhof P, Vereecke N, Stroobants V, Oh D, Vanhee M, Naesens LMJ, Nauwynck HJ. 2021. Functional analysis of human and feline coronavirus cross-reactive antibodies directed against the SARS-CoV-2 fusion peptide. Front Immunol 12:790415. doi:10.3389/fimmu.2021.790415. 35069571 PMC8766817

[21] Voss C, Esmail S, Liu X, Knauer MJ, Ackloo S, Kaneko T, Lowes L, Stogios P, Seitova A, Hutchinson A, Yusifov F, Skarina T, Evdokimova E, Loppnau P, Ghiabi P, Haijan T, Zhong S, Abdoh H, Hedley BD, Bhayana V, Martin CM, Slessarev M, Chin-Yee B, Fraser DD, Chin-Yee I, Li SS. 2021. Epitope-specific antibody responses differentiate COVID-19 outcomes and variants of concern. JCI Insight 6:e148855. doi:10.1172/jci.insight.148855. PMC8410046 34081630

[22] Garrett ME, Galloway JG, Wolf C, Logue JK, Franko N, Chu HY, Matsen FAt, Overbaugh JM. 2022. Comprehensive characterization of the antibody responses to SARS-CoV-2 Spike protein finds additional vaccine-induced epitopes beyond those for mild infection. Elife 11:e73490. doi:10.7554/eLife.73490. 35072628 PMC8887901

[23] Garrett ME, Galloway J, Chu HY, Itell HL, Stoddard CI, Wolf CR, Logue JK, McDonald D, Weight H, Matsen FAt, Overbaugh J. 2021. High-resolution profiling of pathways of escape for SARS-CoV-2 spike-binding antibodies. Cell 184:2927–2938.E11. doi:10.1016/j.cell.2021.04.045. 34010620 PMC8096189

[24] Schaefer SL, Jung H, Hummer G. 2021. Binding of SARS-CoV-2 fusion peptide to host endosome and plasma membrane. J Phys Chem B 125:7732–7741. doi:10.1021/acs.jpcb.1c04176. 34255499 PMC8311640

[25] Coutard B, Valle C, de Lamballerie X, Canard B, Seidah NG, Decroly E. 2020. The spike glycoprotein of the new coronavirus 2019-nCoV contains a furin-like cleavage site absent in CoV of the same clade. Antiviral Res 176:104742. doi:10.1016/j.antiviral.2020.104742. 32057769 PMC7114094

[26] Millet JK, Whittaker GR. 2014. Host cell entry of Middle East respiratory syndrome coronavirus after two-step, furin-mediated activation of the spike protein. Proc Natl Acad Sci USA 111:15214–15219. doi:10.1073/pnas.1407087111. 25288733 PMC4210292

[27] Tang T, Bidon M, Jaimes JA, Whittaker GR, Daniel S. 2020. Coronavirus membrane fusion mechanism offers a potential target for antiviral development. Antiviral Res 178:104792. doi:10.1016/j.antiviral.2020.104792. 32272173 PMC7194977

[28] Bertram S, Dijkman R, Habjan M, Heurich A, Gierer S, Glowacka I, Welsch K, Winkler M, Schneider H, Hofmann-Winkler H, Thiel V, Pöhlmann S. 2013. TMPRSS2 activates the human coronavirus 229E for cathepsin-independent host cell entry and is expressed in viral target cells in the respiratory epithelium. J Virol 87:6150–6160. doi:10.1128/JVI.03372-12. 23536651 PMC3648130

[29] Essalmani R, Jain J, Susan-Resiga D, Andréo U, Evagelidis A, Derbali RM, Huynh DN, Dallaire F, Laporte M, Delpal A, Sutto-Ortiz P, Coutard B, Mapa C, Wilcoxen K, Decroly E, Nq Pham T, Cohen ÉA, Seidah NG. 2022. Distinctive roles of furin and TMPRSS2 in SARS-CoV-2 infectivity. J Virol 96:e0012822. doi:10.1128/jvi.00128-22. 35343766 PMC9044946

[30] Schneider MA, Richtmann S, Grunding AR, Wrenger S, Welte T, Meister M, Kriegsmann M, Winter H, Muley T, Janciauskiene S. 2022. Transmembrane serine protease 2 is a prognostic factor for lung adenocarcinoma. Int J Oncol 60:39. doi:10.3892/ijo.2022.5329. 35211754 PMC8878627

[31] Martin CE, Murray AS, Sala-Hamrick KE, Mackinder JR, Harrison EC, Lundgren JG, Varela FA, List K. 2021. Posttranslational modifications of serine protease TMPRSS13 regulate zymogen activation, proteolytic activity, and cell surface localization. J Biol Chem 297:101227. doi:10.1016/j.jbc.2021.101227. 34562451 PMC8503615

[32] Murray AS, Varela FA, Hyland TE, Schoenbeck AJ, White JM, Tanabe LM, Todi SV, List K. 2017. Phosphorylation of the type II transmembrane serine protease, TMPRSS13, in hepatocyte growth factor activator inhibitor-1 and -2-mediated cell-surface localization. J Biol Chem 292:14867–14884. doi:10.1074/jbc.M117.775999. 28710277 PMC5592667

[33] Hoffmann M, Arora P, Groß R, Seidel A, Hörnich BF, Hahn AS, Krüger N, Graichen L, Hofmann-Winkler H, Kempf A, Winkler MS, Schulz S, Jäck H-M, Jahrsdörfer B, Schrezenmeier H, Müller M, Kleger A, Münch J, Pöhlmann S. 2021. SARS-CoV-2 variants B.1.351 and P.1 escape from neutralizing antibodies. Cell 184:2384–2393.e12. doi:10.1016/j.cell.2021.03.036. 33794143 PMC7980144

[34] Laporte M, Stevaert A, Raeymaekers V, Boogaerts T, Nehlmeier I, Chiu W, Benkheil M, Vanaudenaerde B, Pohlmann S, Naesens L. 2019. Hemagglutinin cleavability, acid stability, and temperature dependence optimize influenza B virus for replication in human airways. J Virol 94:e01430-19. doi:10.1128/JVI.01430-19. 31597759 PMC6912116

[35] Laporte M, Naesens L. 2017. Airway proteases: an emerging drug target for influenza and other respiratory virus infections. Curr Opin Virol 24:16–24. doi:10.1016/j.coviro.2017.03.018. 28414992 PMC7102789

[36] Lucas JM, Heinlein C, Kim T, Hernandez SA, Malik MS, True LD, Morrissey C, Corey E, Montgomery B, Mostaghel E, Clegg N, Coleman I, Brown CM, Schneider EL, Craik C, Simon JA, Bedalov A, Nelson PS. 2014. The androgen-regulated protease TMPRSS2 activates a proteolytic cascade involving components of the tumor microenvironment and promotes prostate cancer metastasis. Cancer Discov 4:1310–1325. doi:10.1158/2159-8290.CD-13-1010. 25122198 PMC4409786

[37] Okumura Y, Takahashi E, Yano M, Ohuchi M, Daidoji T, Nakaya T, Böttcher E, Garten W, Klenk HD, Kido H. 2010. Novel type II transmembrane serine proteases, MSPL and TMPRSS13, proteolytically activate membrane fusion activity of the hemagglutinin of highly pathogenic avian influenza viruses and induce their multicycle replication. J Virol 84:5089–5096. doi:10.1128/JVI.02605-09. 20219906 PMC2863848

[38] Kim DR, Sharmin S, Inoue M, Kido H. 2001. Cloning and expression of novel mosaic serine proteases with and without a transmembrane domain from human lung. Biochim Biophys Acta 1518:204–209. doi:10.1016/s0167-4781(01)00184-1. 11267681

[39] Lukassen S, Chua RL, Trefzer T, Kahn NC, Schneider MA, Muley T, Winter H, Meister M, Veith C, Boots AW, Hennig BP, Kreuter M, Conrad C, Eils R. 2020. SARS-CoV-2 receptor ACE2 and TMPRSS2 are primarily expressed in bronchial transient secretory cells. EMBO J 39:e105114. doi:10.15252/embj.20105114. 32246845 PMC7232010

[40] Vieira Braga FA, Kar G, Berg M, Carpaij OA, Polanski K, Simon LM, Brouwer S, Gomes T, Hesse L, Jiang J, Fasouli ES, Efremova M, Vento-Tormo R, Talavera-López C, Jonker MR, Affleck K, Palit S, Strzelecka PM, Firth HV, Mahbubani KT, Cvejic A, Meyer KB, Saeb-Parsy K, Luinge M, Brandsma CA, Timens W, Angelidis I, Strunz M, Koppelman GH, van Oosterhout AJ, Schiller HB, Theis FJ, van den Berge M, Nawijn MC, Teichmann SA. 2019. A cellular census of human lungs identifies novel cell states in health and in asthma. Nat Med 25:1153–1163. doi:10.1038/s41591-019-0468-5. 31209336

[41] Wang Y, Song W, Wang J, Wang T, Xiong X, Qi Z, Fu W, Yang X, Chen YG. 2020. Single-cell transcriptome analysis reveals differential nutrient absorption functions in human intestine. J Exp Med 217:e20191130. doi:10.1084/jem.20191130. 31753849 PMC7041720

[42] Baron M, Veres A, Wolock SL, Faust AL, Gaujoux R, Vetere A, Ryu JH, Wagner BK, Shen-Orr SS, Klein AM, Melton DA, Yanai I. 2016. A single-cell transcriptomic map of the human and mouse pancreas reveals inter- and intra-cell population structure. Cell Syst 3:346–360.E4. doi:10.1016/j.cels.2016.08.011. 27667365 PMC5228327

[43] MacParland SA, Liu JC, Ma XZ, Innes BT, Bartczak AM, Gage BK, Manuel J, Khuu N, Echeverri J, Linares I, Gupta R, Cheng ML, Liu LY, Camat D, Chung SW, Seliga RK, Shao Z, Lee E, Ogawa S, Ogawa M, Wilson MD, Fish JE, Selzner M, Ghanekar A, Grant D, Greig P, Sapisochin G, Selzner N, Winegarden N, Adeyi O, Keller G, Bader GD, McGilvray ID. 2018. Single cell RNA sequencing of human liver reveals distinct intrahepatic macrophage populations. Nat Commun 9:4383. doi:10.1038/s41467-018-06318-7. 30348985 PMC6197289

[44] Stewart BJ, Ferdinand JR, Young MD, Mitchell TJ, Loudon KW, Riding AM, Richoz N, Frazer GL, Staniforth JUL, Vieira Braga FA, Botting RA, Popescu DM, Vento-Tormo R, Stephenson E, Cagan A, Farndon SJ, Polanski K, Efremova M, Green K, Del Castillo Velasco-Herrera M, Guzzo C, Collord G, Mamanova L, Aho T, Armitage JN, Riddick ACP, Mushtaq I, Farrell S, Rampling D, Nicholson J, Filby A, Burge J, Lisgo S, Lindsay S, Bajenoff M, Warren AY, Stewart GD, Sebire N, Coleman N, Haniffa M, Teichmann SA, Behjati S, Clatworthy MR. 2019. Spatiotemporal immune zonation of the human kidney. Science 365:1461–1466. doi:10.1126/science.aat5031. 31604275 PMC7343525

[45] Garten W, Braden C, Arendt A, Peitsch C, Baron J, Lu Y, Pawletko K, Hardes K, Steinmetzer T, Böttcher-Friebertshäuser E. 2015. Influenza virus activating host proteases: identification, localization and inhibitors as potential therapeutics. Eur J Cell Biol 94:375–383. doi:10.1016/j.ejcb.2015.05.013. 26095298

[46] Elbe S, Buckland-Merrett G. 2017. Data, disease and diplomacy: GISAID's innovative contribution to global health. Glob Chall 1:33–46. doi:10.1002/gch2.1018. 31565258 PMC6607375

