# Supplementary material for: Impact of SARS-CoV-2 Spike Mutations on Its Activation by TMPRSS2 and the Alternative TMPRSS13 Protease
Source: mBio. 2022 Aug 1;13(4):e01376-22. doi: 10.1128/mbio.01376-22 (PMC9426466; doi:10.1128/mbio.01376-22)
Supplement: FIG S1 [file mbio.01376-22-s0001.pdf]

Supplemental Figure S1

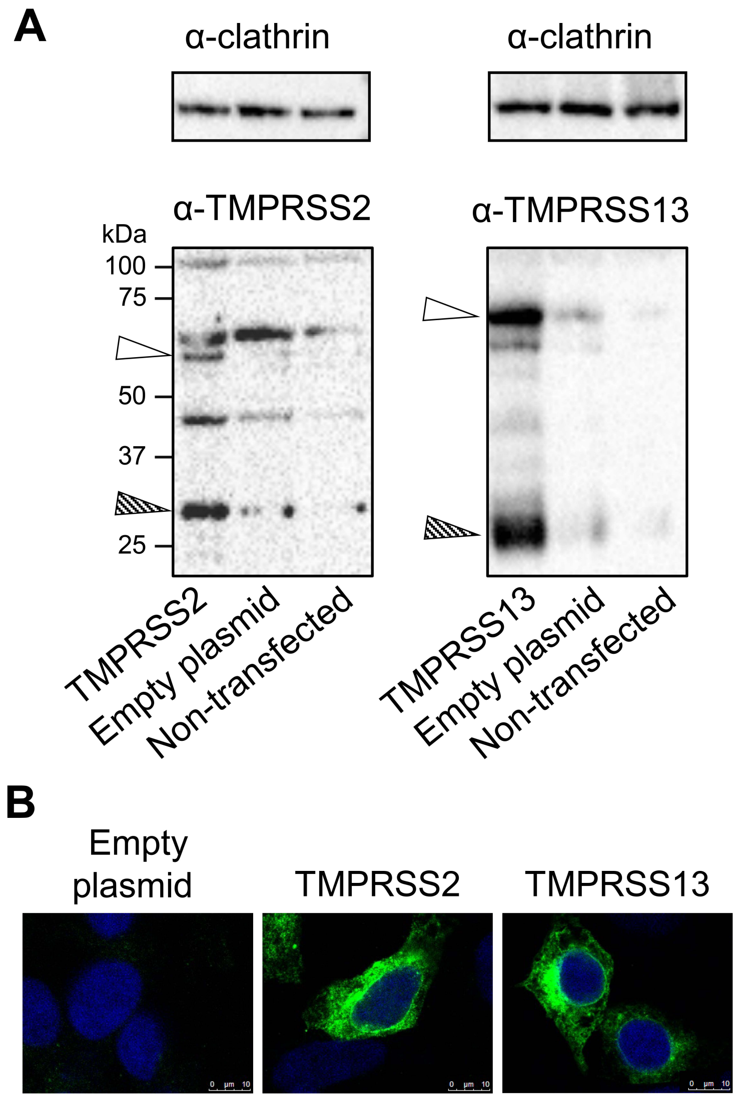

Expression, cleavage and localization of TMPRSS2 and TMPRSS13 following transfection in HEK293T cells. A. Western blot analysis with antibodies directed to the serine protease domain of TMPRSS2 and intracellular region of TMPRSS13. Besides full-length TMPRSS2 and TMPRSS13 (open arrows), autocleavage products (dashed arrows) were prominently present, indicating efficient protease activity. **B.** Confocal microscopy on transfected 293AD cells, stained with anti-flag tag antibody under cell-permeabilized conditions. Scale bar: 10 µm.
