# Supplementary material for: Impact of SARS-CoV-2 Spike Mutations on Its Activation by TMPRSS2 and the Alternative TMPRSS13 Protease
Source: mBio. 2022 Aug 1;13(4):e01376-22. doi: 10.1128/mbio.01376-22 (PMC9426466; doi:10.1128/mbio.01376-22)
Supplement: FIG S2 [file mbio.01376-22-s0002.pdf]

Supplemental Figure S2

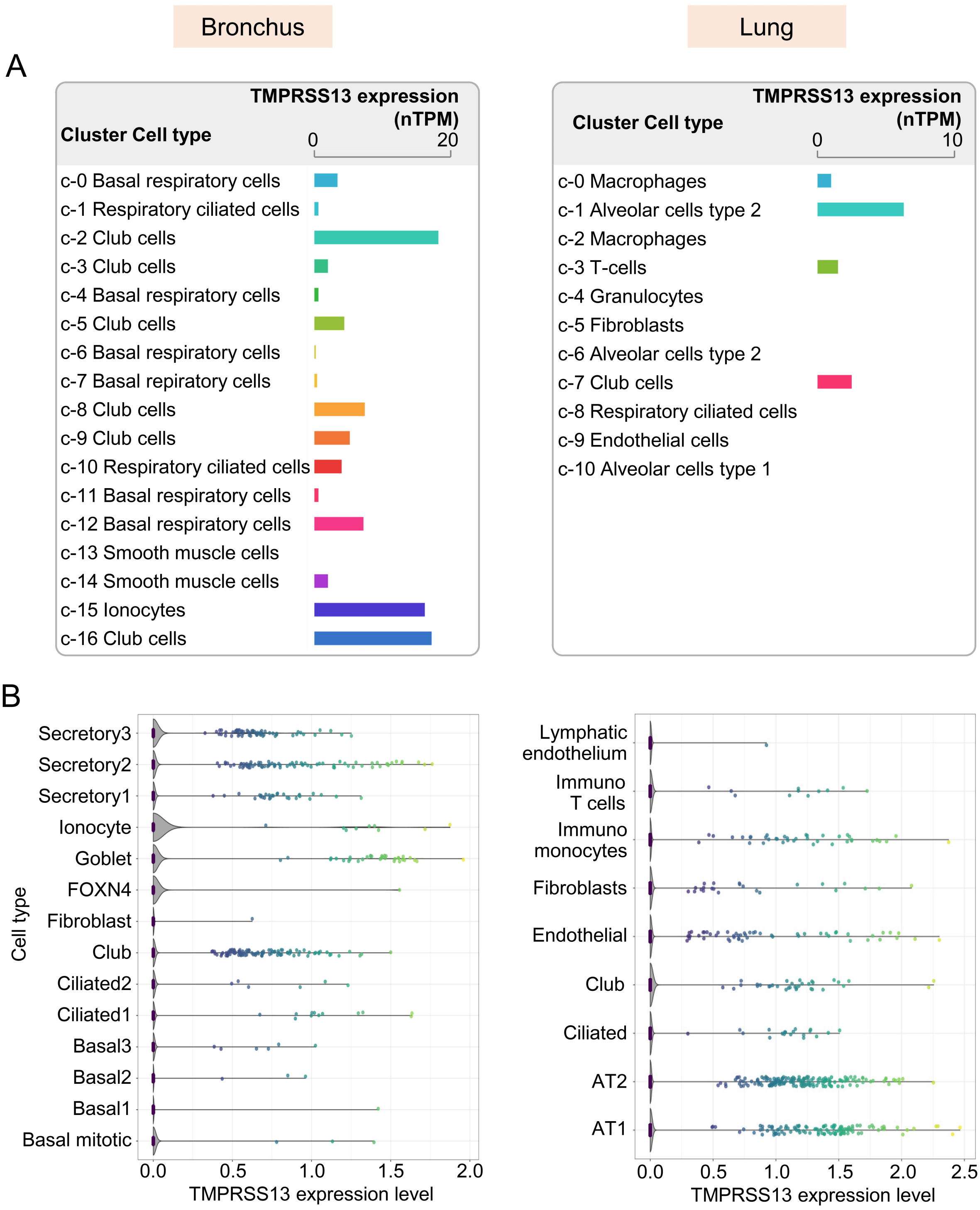

Expression of TMPRSS13 in different cell types of human bronchus (left) and lung (right) tissue. The Figures were made by the open access tools available via: (**A**) Human Protein Atlas proteinatlas.org (1): https://v21.proteinatlas.org/ENSG00000137747-TMPRSS13/single+cell+type/bronchus and https://v21.proteinatlas.org/ENSG00000137747-TMPRSS13/single+cell+type/lung; (**B**) https://digital.bihealth.org/. Panel B shows single-cell RNA sequencing analyses from Lukassen et al. (2) for cells derived from subsegmental bronchial branches (HBEC, human bronchial epithelial cells; left), and lung tissue samples from non-smoking and smoking donors combined (right).

References

1. Karlsson M, Zhang C, Méar L, Zhong W, Digre A, Katona B, Sjöstedt E, Butler L, Odeberg J, Dusart P, Edfors F, Oksvold P, von Feilitzen K, Zwahlen M, Arif M, Altay O, Li X, Ozcan M, Mardinoglu A, Fagerberg L, Mulder J, Luo Y, Ponten F, Uhlén M, Lindskog C. 2021. A single-cell type transcriptomics map of human tissues. Sci Adv 7:eabh2169.

2. Lukassen S, Chua RL, Trefzer T, Kahn NC, Schneider MA, Muley T, Winter H, Meister M, Veith C, Boots AW, Hennig BP, Kreuter M, Conrad C, Eils R. 2020. SARS-CoV-2 receptor ACE2 and TMPRSS2 are primarily expressed in bronchial transient secretory cells. EMBO J 39:e105114.
