# Supplementary material for: Impact of SARS-CoV-2 Spike Mutations on Its Activation by TMPRSS2 and the Alternative TMPRSS13 Protease
Source: mBio. 2022 Aug 1;13(4):e01376-22. doi: 10.1128/mbio.01376-22 (PMC9426466; doi:10.1128/mbio.01376-22)
Supplement: FIG S3 [file mbio.01376-22-s0003.pdf]

Supplemental Figure S3

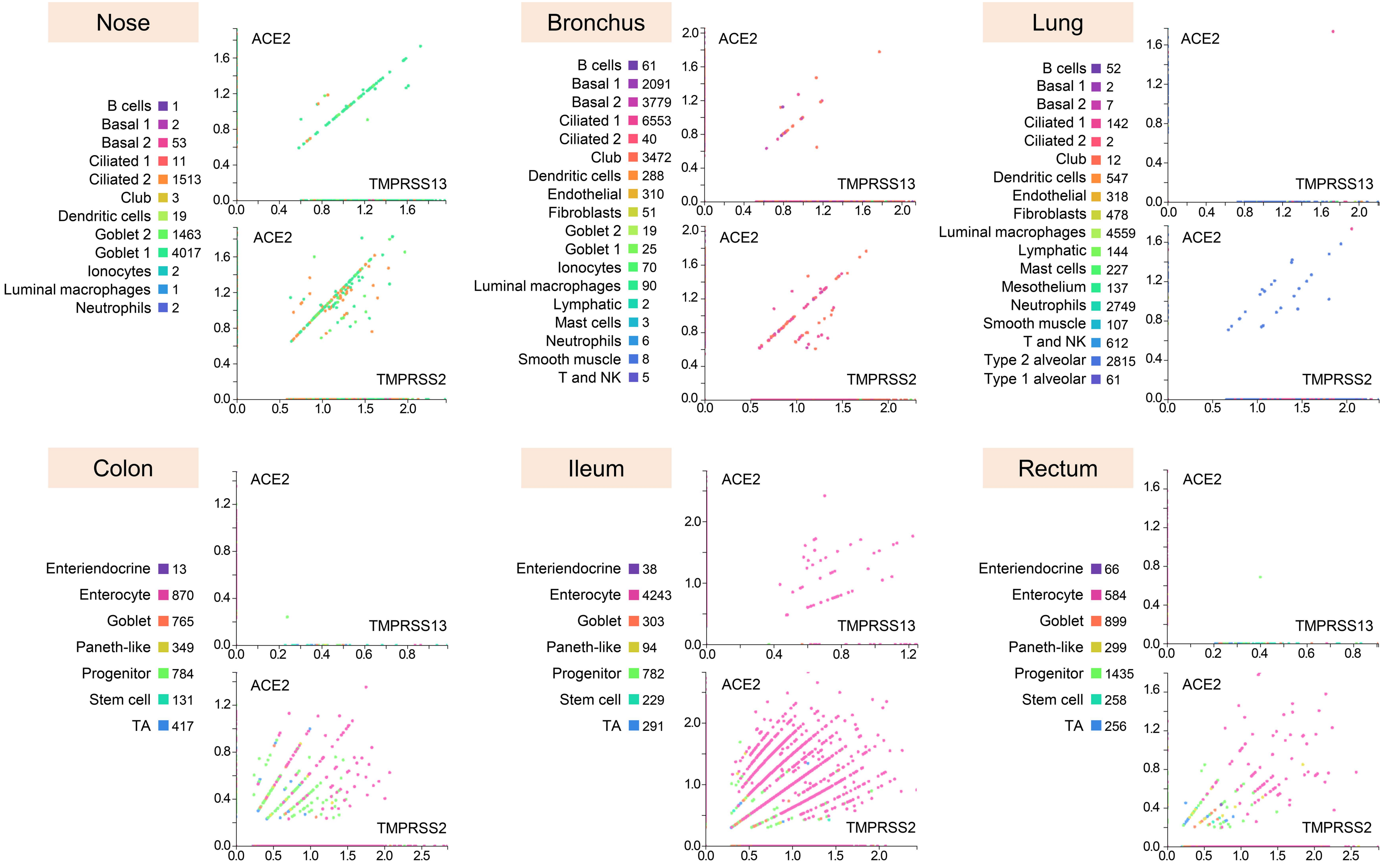

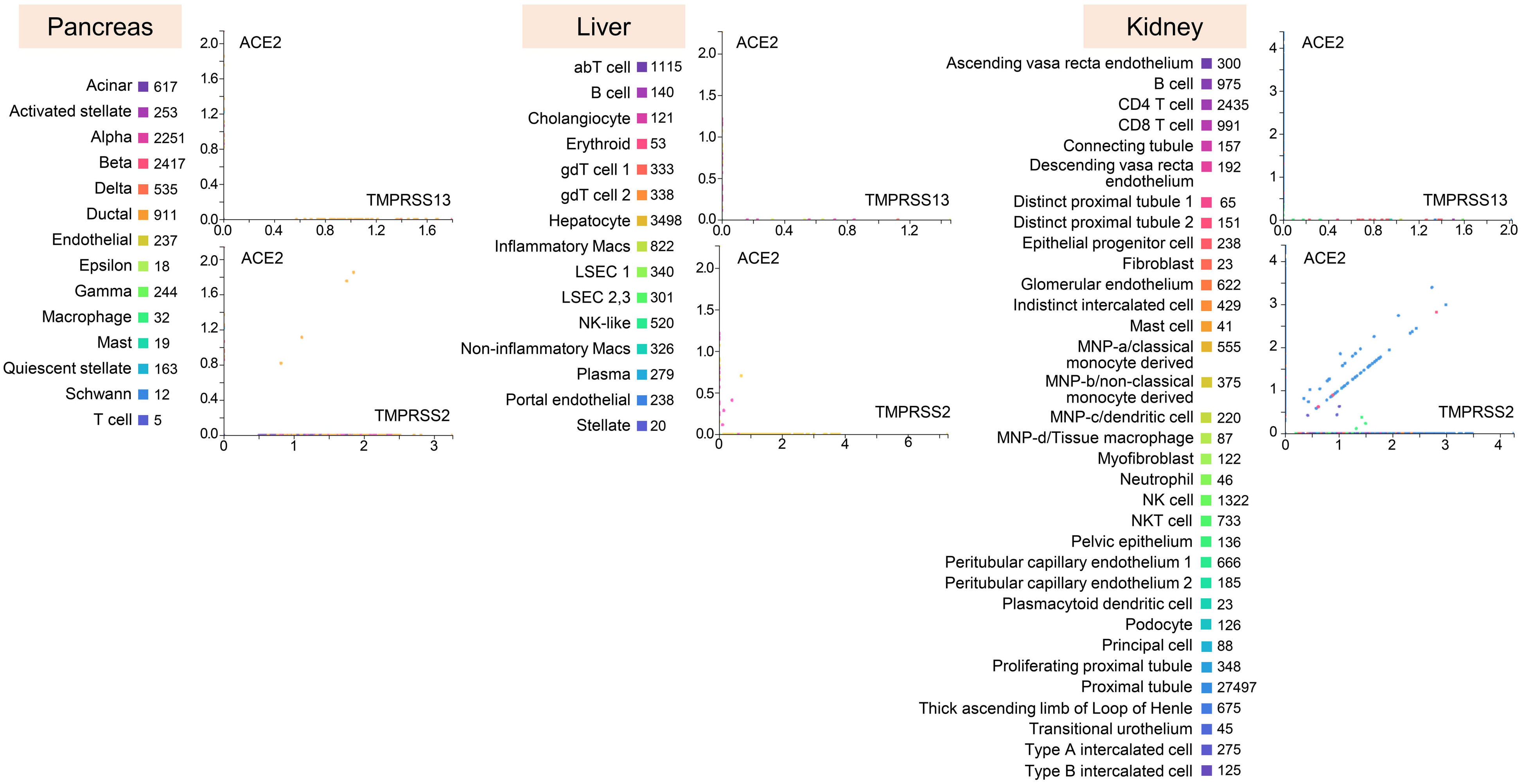

Correlation graphs for *TMPRSS13*, *TMPRSS2* and *ACE2* expression in tissue samples from healthy donors. The dots are colored according to the cell type, specified left to the graphs. These single-cell RNA sequencing analyses are described in the following references: Vieira Braga et al. (1), Wang et al. (2), Baron et al. (3), MacParland et al. (4) and Stewart et al. (5). Graphs were created with https://www.covid19cellatlas.org from Cellxgene Data Portal (6).

References

1. Vieira Braga FA, Kar G, Berg M, Carpaij OA, Polanski K, Simon LM, Brouwer S, Gomes T, Hesse L, Jiang J, Fasouli ES, Efremova M, Vento-Tormo R, Talavera-López C, Jonker MR, Affleck K, Palit S, Strzelecka PM, Firth HV, Mahbubani KT, Cvejic A, Meyer KB, Saeb-Parsy K, Luinge M, Brandsma CA, Timens W, Angelidis I, Strunz M, Koppelman GH, van Oosterhout AJ, Schiller HB, Theis FJ, van den Berge M, Nawijn MC, Teichmann SA. 2019. A cellular census of human lungs identifies novel cell states in health and in asthma. Nat Med 25:1153-1163.

2. Wang Y, Song W, Wang J, Wang T, Xiong X, Qi Z, Fu W, Yang X, Chen YG. 2020. Single-cell transcriptome analysis reveals differential nutrient absorption functions in human intestine. J Exp Med 217:e20191130.

3. Baron M, Veres A, Wolock SL, Faust AL, Gaujoux R, Vetere A, Ryu JH, Wagner BK, Shen-Orr SS, Klein AM, Melton DA, Yanai I. 2016. A single-cell transcriptomic map of the human and mouse pancreas reveals inter- and intra-cell population structure. Cell Syst 3:346-360.e4.

4. MacParland SA, Liu JC, Ma XZ, Innes BT, Bartczak AM, Gage BK, Manuel J, Khuu N, Echeverri J, Linares I, Gupta R, Cheng ML, Liu LY, Camat D, Chung SW, Seliga RK, Shao Z, Lee E, Ogawa S, Ogawa M, Wilson MD, Fish JE, Selzner M, Ghanekar A, Grant D, Greig P, Sapisochin G, Selzner N, Winegarden N, Adeyi O, Keller G, Bader GD, McGilvray ID. 2018. Single cell RNA sequencing of human liver reveals distinct intrahepatic macrophage populations. Nat Commun 9:4383.

5. Stewart BJ, Ferdinand JR, Young MD, Mitchell TJ, Loudon KW, Riding AM, Richoz N, Frazer GL, Staniforth JUL, Vieira Braga FA, Botting RA, Popescu DM, Vento-Tormo R, Stephenson E, Cagan A, Farndon SJ, Polanski K, Efremova M, Green K, Del Castillo Velasco-Herrera M, Guzzo C, Collord G, Mamanova L, Aho T, Armitage JN, Riddick ACP, Mushtaq I, Farrell S, Rampling D, Nicholson J, Filby A, Burge J, Lisgo S, Lindsay S, Bajenoff M, Warren AY, Stewart GD, Sebire N, Coleman N, Haniffa M, Teichmann SA, Behjati S, Clatworthy MR. 2019. Spatiotemporal immune zonation of the human kidney. Science 365:1461-1466.

6. Chanzuckerberg Initiative. Cellxgene Data Portal. Retrieved on 02/07/2022, from https://cellxgene.cziscience.com/.
