# Supplementary material for: Impact of SARS-CoV-2 Spike Mutations on Its Activation by TMPRSS2 and the Alternative TMPRSS13 Protease
Source: mBio. 2022 Aug 1;13(4):e01376-22. doi: 10.1128/mbio.01376-22 (PMC9426466; doi:10.1128/mbio.01376-22)
Supplement: FIG S4 [file mbio.01376-22-s0004.pdf]

Supplemental Figure S4

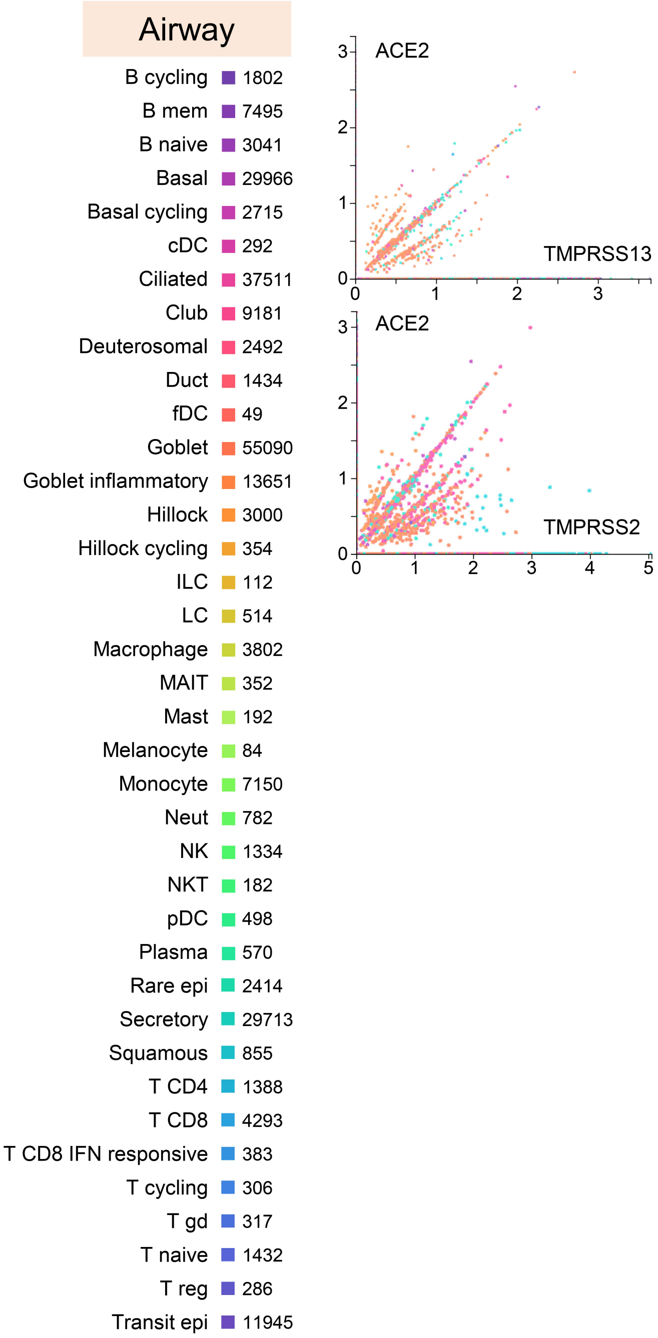

Correlation graphs for *TMPRSS13*, *TMPRSS2* and *ACE2* expression in airway tissue samples from COVID-19 patient donors. Graphs created with https://www.covid19cellatlas.org from Cellxgene Data Portal (Chanzuckerberg Initiative; https://cellxgene.cziscience.com/), the dots are colored according to the cell type, specified left to the graphs.
